# Highly Conducting Li(Fe_1−*x*_Mn_*x*_)_0.88_V_0.08_PO_4_ Cathode Materials Nanocrystallized from the Glassy State (*x* = 0.25, 0.5, 0.75)

**DOI:** 10.3390/ma14216434

**Published:** 2021-10-27

**Authors:** Justyna E. Frąckiewicz, Tomasz K. Pietrzak

**Affiliations:** Faculty of Physics, Warsaw University of Technology, Koszykowa 75, PL-00-662 Warsaw, Poland; frackiewicz.justyna95@gmail.com

**Keywords:** nanocrystallization, high conductivity, electron hopping, olivine, cathode materials

## Abstract

This study showed that thermal nanocrystallization of glassy analogs of LiFe${}_{1-x}$Mn${}_{x}$PO${}_{4}$ (with the addition of vanadium for improvement of glass forming properties) resulted in highly conducting materials that may be used as cathodes for Li-ion batteries. The glasses and nanomaterials were studied with differential thermal analysis, X-ray diffractometry, and impedance spectroscopy. The electrical conductivity of the nanocrystalline samples varied, depending on the composition. For x=0.5, it exceeded $10^{-3}$ S/cm at room temperature with an activation energy as low as 0.15 eV. The giant and irreversible increase in the conductivity was explained on the basis of Mott’s theory of electron hopping and a core-shell concept. Electrochemical performance of the active material with x=0.5 was also reported.

## 1. Introduction

Lithium batteries have been developed for more that 70 years [1]. However, the electrochemical properties of lithium itself were studied even earlier, in 1913 [2]. Since the late 1960s, non-aqueous 3-V lithium-ion primary batteries have been available in the market. In 1974, M.S. Whittingham patented the Li//TiS${}_{2}$ battery. In 1973, J.B. Goodenough et al. proposed LiCoO${}_{2}$ as a new cathode with a potential as high as 3.9 V vs. Li${}^{+}$/Li. This opened a new era for the Li-ion battery market. In 1997, J.B. Goodenough et al. [3] proposed a new class of cathode materials—phospho-olivines—and since then LiMPO${}_{4}$ (M = Fe, Mn, Co, Ni) materials have been widely studied for their application [4]. From the whole family of isostructural compounds, only LiFePO${}_{4}$ was successfully introduced into mass production. LiMnPO${}_{4}$ has a significantly higher potential versus metallic lithium compared to LiFePO${}_{4}$ (4.13 V and 3.43 V, respectively) and a comparable theoretical gravimetric capacity ca. 170 mAh/g [4]. However, the synthesis of LiMnPO${}_{4}$ compounds, which can work in batteries with high loads, is more difficult [5]. One of the successful synthetic routes consists in the preparation of intentionally non-stoichiometric compositions [6]. Another possible method is to synthesize LiMn${}_{1-x}$Fe${}_{x}$PO${}_{4}$ phospho-olivines [7,8], i.e., LiMnPO${}_{4}$ with partial Mn substitution to Fe.

The low electronic conductivity of LiMPO${}_{4}$ materials belongs to a group of factors that significantly limit their electrochemical performances [4]. This issue is usually addressed by surface coating with a highly conducting nanometer thickness layer or by particle size control. In recent years, J.E. Garbarczyk’s group has proposed and investigated an alternative route to the conductivity enhancement—a thermal nanocrystallization of glassy analogs of selected crystalline cathode materials, such as: V${}_{2}$O${}_{5}$, LiFePO${}_{4}$, and Li${}_{3}$V${}_{2}$(PO${}_{4}$)${}_{3}$ [9]. This approach has several advantages: the absence of carbon additives, simplicity, and the straightforwardness of synthesis. Preparation consists of two stages only: (i) glass preparation by melt-quenching and (ii) proper thermal treatment of the glass to conduct its nanocrystallization. By the appropriate heat treatment, one can achieve a giant (even by a factor $10^{9}$) and irreversible conductivity enhancement.

The possibility and influence of iron partial substitution with vanadium in LiFePO${}_{4}$ was studied, e.g., by M.S. Whittingham and co-workers [10]. It was demonstrated that the addition of vanadium enhances the electrochemical performance of the materials, especially at high current densities. From the point of view of thermal nanocrystallization, the addition of vanadium also improves glass-forming properties of the compound and positively affects the electronic hopping. Such an effect was observed in LiFe${}_{x}$V${}_{1-2.5x}$PO${}_{4}$ glasses and nanomaterials [11]. In recent research [12], we aimed to replace some of the iron ions in LiFe${}_{0.88}$V${}_{0.08}$PO${}_{4}$ glass with manganese in order to obtain highly conducting nanomaterials. The reasons for introducing some vacancies on Fe sites are as follows. Firstly, this provides charge compensation, when Fe${}^{2+}$ ions are replaced by V${}^{3+}$ ions. Secondly, nonstoichiometry may lead to an improvement in electrochemical performance, as reported, e.g., in [13].

In this article, extended studies on three compositions Li(Fe${}_{1-x}$Mn${}_{x}$)${}_{0.88}$V${}_{0.08}$PO${}_{4}$ with different Fe and Mn contents are reported. In particular, we focused on the influence of the Fe/Mn ratio on the electrical conductivity of synthesized nanomaterials.

## 2. Materials and Methods

Three compositions of general formula Li(Fe${}_{1-x}$Mn${}_{x}$)${}_{0.88}$V${}_{0.08}$PO${}_{4}$ (x = 0.25, 0.5, 0.75) were selected for investigation (Table 1). Appropriate amounts of precursors: Li${}_{2}$CO${}_{3}$, FeC${}_{2}$O${}_{4}\cdot$2H${}_{2}$O, Mn(CH${}_{3}$COO)${}_{2}\cdot$4H${}_{2}$O, (NH${}_{4}$)H${}_{2}$PO${}_{4}$, and V${}_{2}$O${}_{5}$ were mixed in a mortar, melted at 1300 °C in a reducing atmosphere, and rapidly quenched. Their amorphousness was verified with X-ray diffractometry (XRD). Thermal events occurring in the samples were observed with differential thermal analysis (DTA), using a SDT Q600 setup (TA Instruments). The measurements were conducted with a heating rate of 10 °C/min in argon flow. Crystallization processes occurring upon heating were observed by HT-XRD in nitrogen flow, preventing the samples from possible oxidation. The diffraction studies were carried out on a Philips X’Pert Pro apparatus using the Cu${}_{K\alpha }$ line (λ = 1.542 Å), equipped with an Anton Paar oven. Electrical conductivity was measured upon heating and subsequent cooling ramps with impedance spectroscopy within the wide frequency range 10 mHz–10 MHz. The set-up consisted of a Novocontrol Alfa-A analyzer and a tube furnace (Czylok) controlled by Eurotherm 2404 [14]. The spectra were acquired when a temperature stability as good as 0.1 °C was reached. The step between measurements was 25 °C. The average heating/cooling rate was less than 1 °C/min. For this experiment, platinum electrodes were sputtered at the opposite sides of the studied samples.

The sample with x=0.5 was selected for electrochemical characterization. About 1 g of the sample was heated in a tube furnace to 580 °C at 1 °C/min heating rate, i.e., in conditions similar to these used in the electrical measurements, in order to obtain a highly-conducting material. The procedure was carried out in argon flow to prevent the material from oxidation. Then, the sample was mixed with carbon black and ball-milled in a planetary mill for 20 hrs at 300 rpm in order to get a fine powder. To prepare the layer (75 wt% active material, 15 wt% carbon black (CB–TIMCAL Graphite & Carbon Super P^®^ Conductive Carbon Black), and 10 wt% PVDF), a slurry was made by mixing these materials in N-methyl-2-pyrrolidone (NMP, Aldrich) for 3 h, in order to obtain a homogenous mixture, using a magnetic stirrer. The suspension was spread at room temperature on an aluminium current collector by using a doctor blade. The gap was set to 0.1 mm. After the evaporation of the solvent in an oven at 50 °C for 24 h, the foil was transferred to an Ar-filled dry-box, where the procedure continued. It was cut in disks of 12 mm in diameter with a loading of the active material of about 3 mg/cm${}^{2}$. A metallic lithium plate was used as an anode and 1 M LiPF${}_{6}$ (in EC : DEC) as the liquid electrolyte. The cell was charged/discharged with rates varying from C/50 to C within the 2.0–4.5 V range.

Cyclic voltammetry (CV) was performed in a three-electrode Swagelok-type cell with the prepared cathode layer as a working electrode, metallic lithium plates as the reference and the counter electrodes, and with a liquid electrolyte. Firstly, the cell was held at an open circuit voltage (OCV) for stabilization for 24 h and then measured in the potential range of 2.0–4.7 V (vs. Li${}^{+}$/Li${}^{0}$) at a scan rate of 0.05 mV/s in 5 cycles.

As a supplementary study, the microstructure of the sample with x=0.5 was investigated with a high-resolution transmission electron microscopy (HR-TEM). It was performed using a FEI Titan Cubed 80-300 microscope at the Institute of Physics, Polish Academy of Sciences.

## 3. Results and Discussion

### 3.1. Differential Thermal Analysis (DTA)

**Figure 1:**
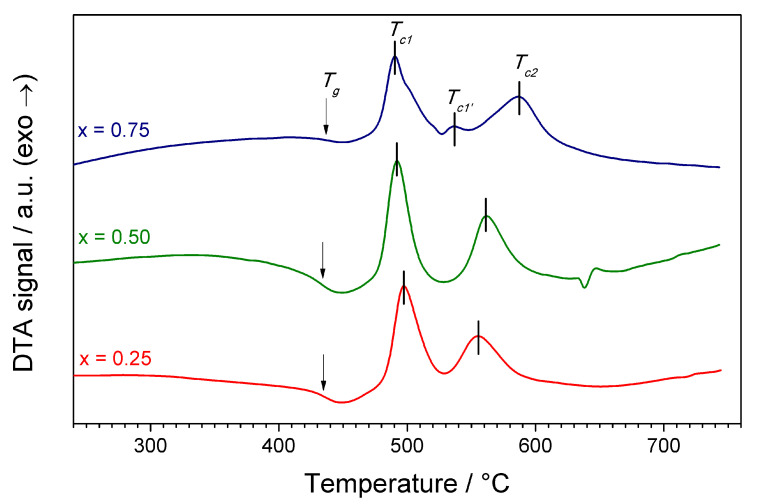
DTA curves for Li(Fe${}_{1-x}$Mn${}_{x}$)${}_{0.88}$V${}_{0.08}$PO${}_{4}$ glasses measured in argon flow with a heating rate 10 °C/min. © The Electrochemical Society. Reproduced from Ref. [12] by permission of IOP Publishing. All rights reserved.

DTA curves of the synthesized samples were typical for glassy materials (Figure 1). A glass transition and two or three crystallization peaks were observed. The glass transition temperature was ca. 435 °C regardless the composition. The main crystallization peak was centered at ca. 490 °C. The position of the second significant crystallization peak varied from 555 to 587 °C and was shifted towards higher temperatures for the samples with greater manganese content. In the sample with x=0.75, an additional minor crystallization peak appeared at 540 °C. In the sample with x=0.5, an endothermal event was observed at ca 650 °C. The origin of this event is unclear. It may be a melting of one of the phases, and it determined the upper thermal stability of crystallized materials. Additionally, in Figure 1, one can see that the ratio between areas of the first and the second peak became smaller with increasing *x*.

Exact temperatures of the observed thermal events are presented in Table 2. The temperature of the first crystallization peak decreased with the increasing Mn content, whereas the temperature of the second crystallization peak significantly increased with the increasing value of *x*. This may be related to the energy of formation LFP and LMP phases upon different concentrations of iron and manganese. Differences in crystallization temperatures were previously observed e.g., in the case of LiFeBO${}_{3}$ and LiMnBO${}_{3}$ glasses [15,16].

### 3.2. X-ray Diffractometry (XRD)

**Figure 2:**
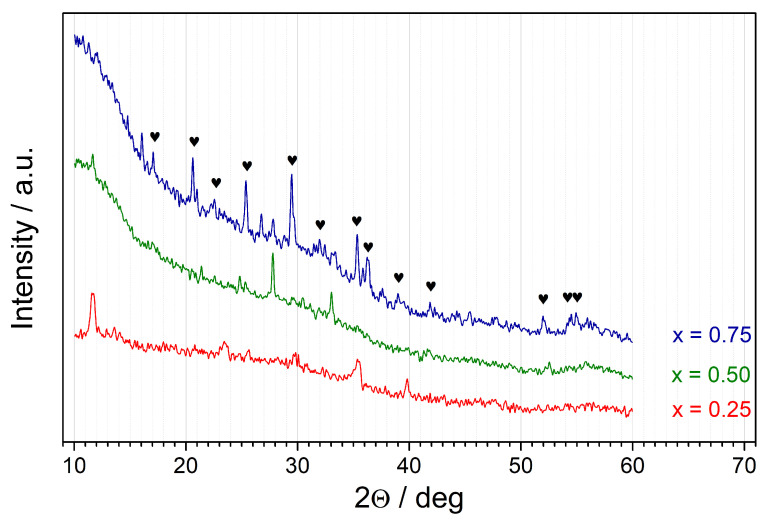
XRD patterns for synthesized Li(Fe${}_{1-x}$Mn${}_{x}$)${}_{0.88}$V${}_{0.08}$PO${}_{4}$ samples measured at room temperature. Diffraction lines ascribed to Li(Fe${}_{0.25}$Mn${}_{0.75}$)PO${}_{4}$ reference pattern (ICDD card no. 04-024-8018) are marked with hearts.

While DTA curves were typical for glassy materials, XRD patterns of the synthesized samples (Figure 2) appeared to be intriguing. One can observe an amorphous halo at low angles (20–40°). However, low-intensity but distinct peaks were observable in all samples. This means that the samples had partially crystallized upon fast cooling from the melt. In the case of samples with x≤0.5, the identification of crystalline phases was difficult, due to the low intensity of the peaks. In the case of x=0.75, the positions of the major peaks were in agreement with Li(Fe${}_{0.25}$Mn${}_{0.75}$)PO${}_{4}$ reference pattern (ICDD card no. 04-024-8018).

**Figure 3:**
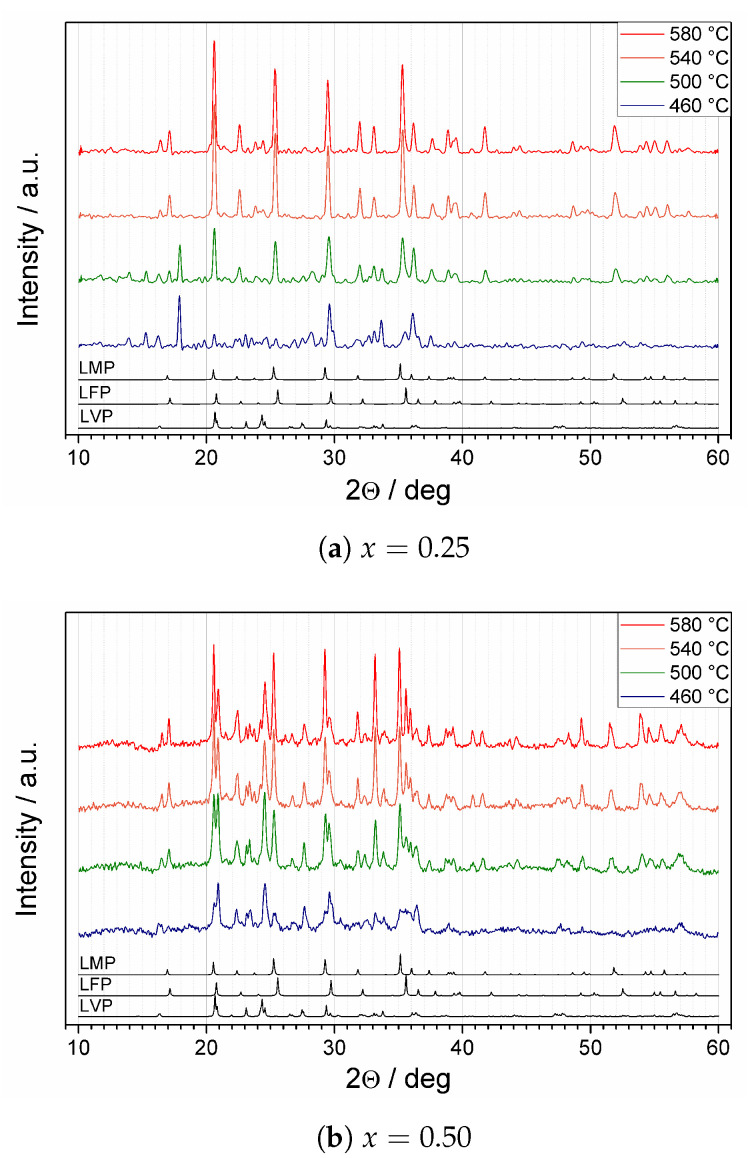

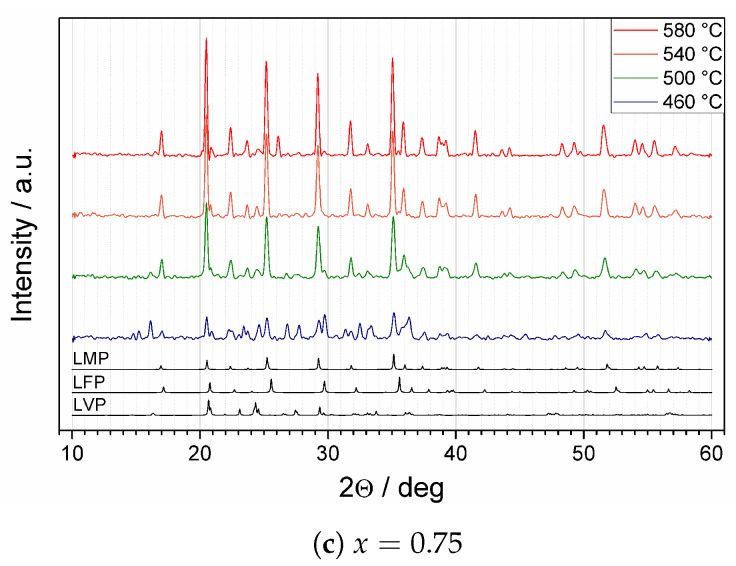
Temperature-dependent XRD patterns for Li(Fe${}_{1-x}$Mn${}_{x}$)${}_{0.88}$V${}_{0.08}$PO${}_{4}$ samples upon heating to 580 °C in nitrogen flow. Reference patterns for LiFePO${}_{4}$ (LFP), LiMnPO${}_{4}$ (LMP), and Li${}_{3}$V${}_{2}$(PO${}_{4}$)${}_{3}$ (LVP) are given below. Figure (b) reproduced from Ref. [12] by permission of IOP Publishing. © The Electrochemical Society. All rights reserved.

Regardless of the initial impurities, XRD patterns acquired upon heating the samples to 580 °C (Figure 3a–c) confirmed crystallization in three crystalline phases: triphylite LiFePO${}_{4}$ (abbrev. LFP, space group Pnma), lithiophilite LiMnPO${}_{4}$ (abbrev. LMP, space group Pmnb), and lithium vanadium phosphate Li${}_{3}$V${}_{2}$(PO${}_{4}$)${}_{3}$ (abbrev. LVP, space group $P2_{1}/n$). Since the positions of the peaks in all three patterns were quite similar and the peaks in nanocrystalline samples were broad, it was not easy to distinguish the crystallization of each phase at first sight. In general, the unit cell constants of LiFe${}_{1-x}$Mn${}_{x}$PO${}_{4}$ increases with increasing manganese content [17]. Therefore the diffraction lines of LMP were shifted towards lower Braggs’ angles, in comparison to LFP. The quality of the patterns and their complexity did not allow us to perform reliable Rietveld refinement. Nevertheless, an analysis of minor peaks allowed us to suspect that the crystallization of Li${}_{3}$V${}_{2}$(PO${}_{4}$)${}_{3}$ appeared first and was followed by LiFePO${}_{4}$. Due to lower vanadium content, these two processes might overlap in the first crystallization peak observed by DTA. Therefore the second crystallization peak can be ascribed to the crystallization of LiMnPO${}_{4}$. This hypothesis is supported by the fact that the ratio of the areas under these two peaks becomes a favorite for the second crystallization process with growing *x*, i.e., with growing Mn content in the nominal composition. Eventually, most of the the reflexes originating from LFP and LMP merged at high temperatures.

**Figure 4:**
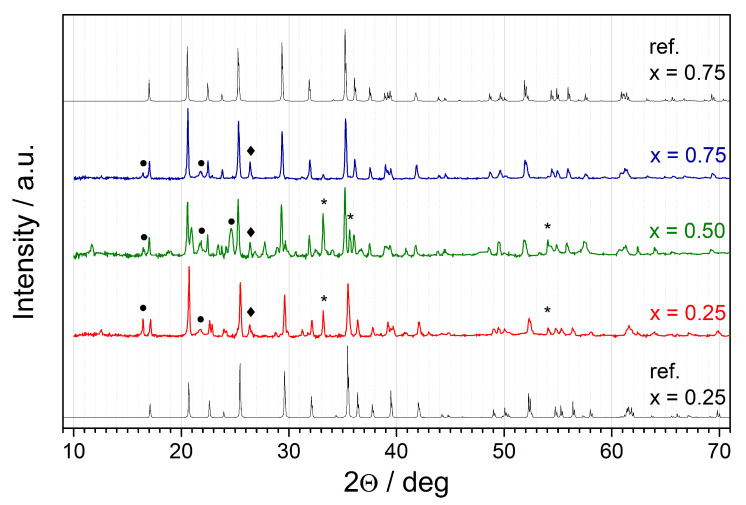
A comparison of the room temperature XRD patterns after heat-treatment at 580 °C for x=0.25, 0.5, and 0.75. Reference patterns of Li(Fe${}_{0.75}$Mn${}_{0.25}$)PO${}_{4}$ (x=0.25, at the bottom, ICDD card no. 00-066-0406) and Li(Fe${}_{0.25}$Mn${}_{0.75}$)PO${}_{4}$ (x=0.75, at the top, ICDD card no. 04-024-8018) are provided for comparison. Major reflexes assigned to impurity phases are marked as follows: circle–Li${}_{3}$V${}_{2}$(PO${}_{4}$)${}_{3}$ (CIF no. 4124523), asterisk–Fe${}_{2}$O${}_{3}$ (ICSD card no. 98-005-6372), diamond–V${}_{2}$O${}_{5}$ (ICDD card no. 04-006-5671).

In Figure 4, one can see a comparison between three high-resolution patterns collected at room temperature after heat treatment for each value of *x*. The reference patterns for mixed LiFe${}_{1-x}$Mn${}_{x}$PO${}_{4}$ compounds with x=0.25 and 0.75 are given as well. One can see that the position of the main peaks slightly shifts towards lower angles, and it is in good agreement with the reference patterns. It suggests that for compositions with x=0.25 and 0.75, Mn/Fe ions incorporate into the same structure with different unit-cell parameters. On the contrary, for the composition with x=0.5, separated lines from iron-rich and manganese-rich olivine-like phases were observed. Some impurity phases were also detected and identified, including Fe${}_{2}$O${}_{3}$, V${}_{2}$O${}_{5}$, and Li${}_{3}$V${}_{2}$(PO${}_{4}$)${}_{3}$. This suggests that not all of vanadium was doped into the olivine structure.

### 3.3. Electrical Conductivity

**Figure 5:**
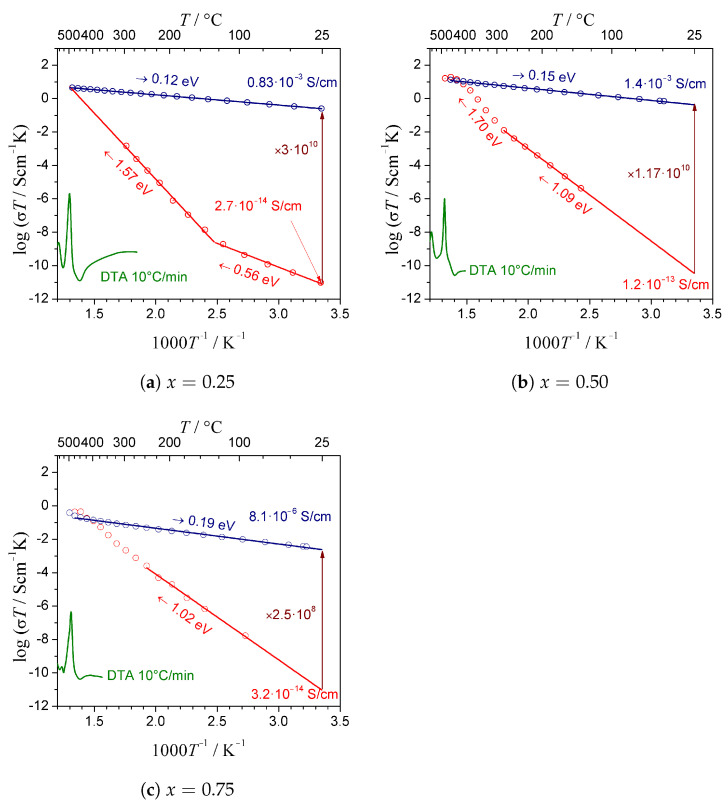
Dependence of electrical conductivity of samples with *x* = 0.25, 0.5, and 0.75 upon heating to 480 °C (red ramps) and subsequent cooling down to room temperature (blue ramps). The red and blue values of the conductivity were measured at 25 °C and are given for the starting materials and the samples after nanocrystallization, respectively. Corresponding DTA curves (green lines, shown in arbitrary units) measured with heating rate 10 °C/min are given for comparison.

The initial electrical conductivity of as-synthesized glassy samples at room temperature was modest, within the $10^{-14}$–$10^{-13}$ S/cm range. The impedance figures in Nyquist coordinates were similar for all compositions and consisted of a single semicircle, which is a typical shape for glasses with predominant electronic conductivity. In the glassy phase, the Li${}^{+}$ conductivity might be suppressed by a lack of conduction channels that are present in a periodic crystalline structure. However, it was a good starting point for significant improvement. IS measurements performed for the samples showed that a proper thermal treatment of glassy samples resulted in a significant and irreversible increase in the conductivity (Figure 5a–c). The best electrical conductivity of a nanocrystallized sample—i.e., $1.4\cdot 10^{-3}$ S/cm at RT—was observed for the composition with x=0.5. A slightly lower value—i.e., $0.8\cdot 10^{-3}$ S/cm at RT—was recorded for the composition with x=0.25. The lowest value—below $10^{-5}$ S/cm at RT—was reached in the case of x=0.75. The values of the activation energy ranged from 0.12 eV to 0.19 eV for x=0.25 and x=0.75, respectively. These values were much better than the electronic conductivity in LiFePO${}_{4}$ crystals, which was $10^{-7}$ S/cm at room temperature, and its activation energy varied between 0.55 and 0.59 eV [18].

**Figure 6:**
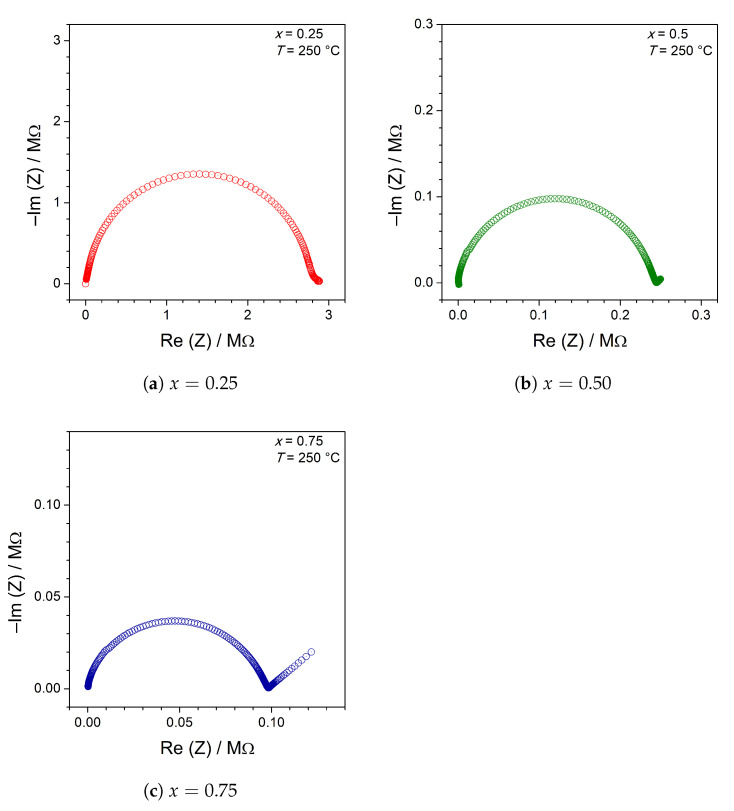
Examples of impedance (Nyquist) plots for selected samples acquired at isothermal conditions at ca. 250 °C upon the heating ramp.

The differences in electrical properties appeared also at elevated temperatures. Imped-ance figures acquired at ca. 250 °C presented in Nyquist plots are shown in Figure 6a–c. Mainly, they consisted of a single semicircle. The equivalent circuit can be described as (RP), where *R* is the total resistance of the samples and *P* is a constant phase element (CPE), with parameter *n* close to 1. However, for samples with x≥0.5, an ionic spur at low frequencies was more pronounced. This behavior can be modeled with a serial CPE element with n≈0.5. More detailed discussion of basic equivalent circuits describing electronic and ionic conductors can be found, e.g., in Ref. [19].

Upon cooling, the impedance figures were strongly affected by the induction of a holder, due to low resistance of the samples. Nevertheless, no low-frequency spur was observed, which would be evidence for ionic conductivity with values comparable to electronic conduction. This is not to say that nanocrystalline materials exhibited no ionic conductivity, but it had to be a couple of orders of magnitude lower than the very high electronic conductivity of nanocrystallized samples.

Such a behavior has been previously observed by us in many glassy analogs of cathode materials, e.g., olivine-like ones [9,20]. Such a phenomenal increase in the electrical conductivity and a significant decrease in the activation energy can be explained on the basis of Mott’s theory of electron hopping in oxide glasses containing transition metal ions (i.e., Fe, Mn, and V) [21]. In our approach, the conductivity increase can be ascribed to the formation of interfacial regions (shells) around the nanocrystallites (cores). The resulting mixed valence of iron, manganese, and vanadium in these regions is advantageous for small polaron hopping, because the distances between pairs of hopping centers, (Fe${}^{2+}$–Fe${}^{3+}$, Mn${}^{2+}$–Mn${}^{3+}$, and V${}^{3+}$–V${}^{4+}$), becomes shorter. A detailed explanation of this phenomenon and further discussion of the core-shell concept can be found, e.g., in Ref. [9]. At this point, it is worth mentioning that alternative hypotheses for the giant increase in the conductivity were carefully investigated and, eventually, rejected. This phenomenon cannot be ascribed to the appearance of metallic easy conductive paths as in the work: [22]. The increase in the conductivity due to metal-insulator transition in vanadium oxides does not explain the phenomenon, as this transition would be reversible in a function of the temperature [23].

### 3.4. Electrochemical Characterization

**Figure 7:**
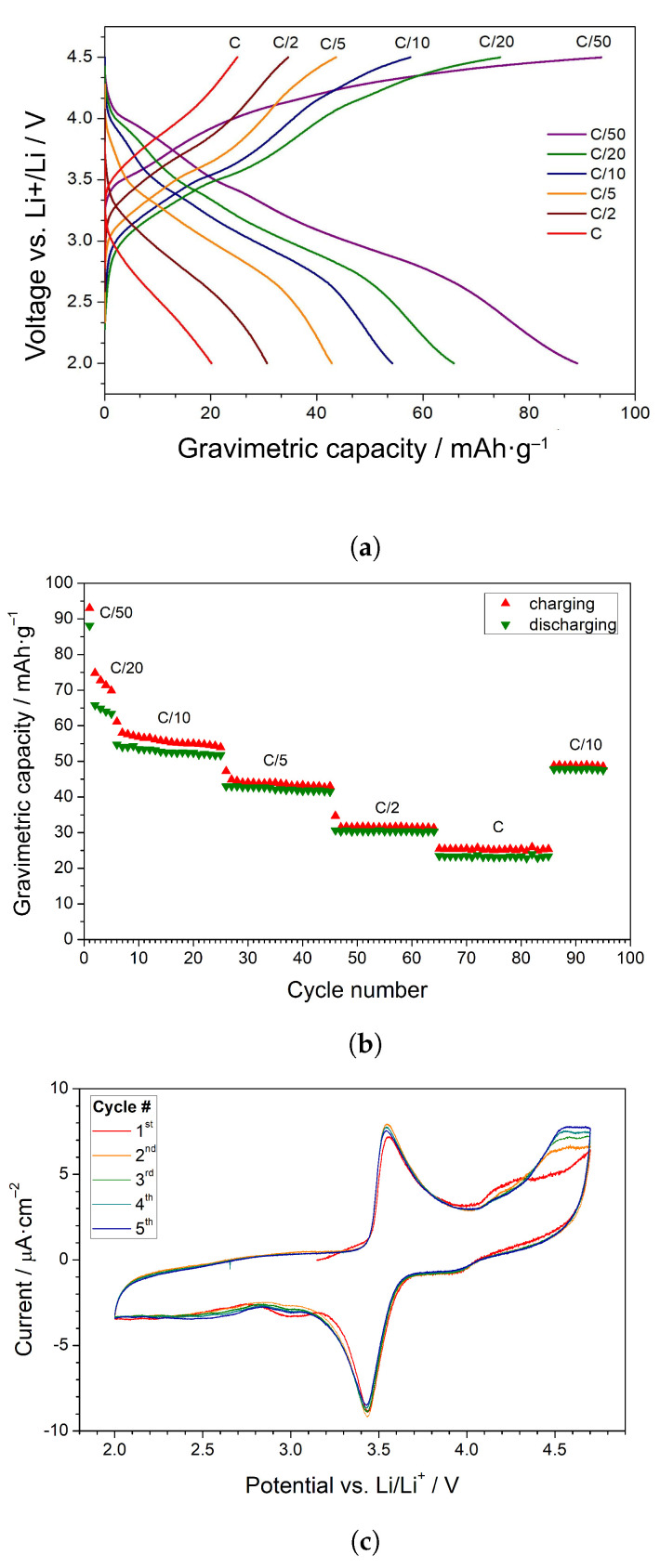
(**a**) Charge/discharge curves of a lithium cell made of the nanocrystallized sample with *x* = 0.5 measured at different rates. (**b**) Cyclability of the cell. (**c**) Cyclic voltammogram of the cell for 5 cycles measured at the scanning rate of 0.05 mV/s.

In Figure 7a, charge–discharge curves at various current rates from C/50 to C are shown. In general, the potential is monotonically changing. However, two steps are usually observable. One could ascribe Fe${}^{2+}$/Fe${}^{3+}$ and Mn${}^{2+}$/Mn${}^{3+}$ redox pairs to these features. However, typical crystalline olivine cathode materials exhibit a broad plateau, resulting in a nearly constant potential during charging and discharging. A mix LiFe${}_{1-x}$Mn${}_{x}$PO${}_{4}$ compound exhibits a similar behavior, with two plateaus corresponding to iron and manganese redox pairs [17,24,25]. On the contrary, a continuous change in the potential may be evidence for the presence of a non-stoichiometric single phase in nanograins, rather than a two-phase mechanism (i.e., fully lithiated and entirely delithiated phases). Quite similar charge/discharge curves were observed for nano LiFePO${}_{4}$ by P. Gibot et al. [26]. In our experiment, only up to 95 mAh/g was reached with a 4.5-V cutoff, which is considerably lower than the theoretical capacity (ca. 170 mAh/g). The cyclability of the cell is presented in Figure 7b. We may expect that the rest of the capacity could be reached at a higher potential due to the V${}^{3+}$/V${}^{4+}$ redox pair in Li${}_{3}$V${}_{2}$(PO${}_{4}$)${}_{3}$.

In Figure 7c, CV curves of a prepared lithium cell are shown. In all cycles, the highest oxidation peak was observed around 3.6 V, which corresponds to a step observed in the charge curve described earlier. The associated redox peak at a lower potential ca. 3.4 V confirms the appearance of Fe${}^{2+}$/Fe${}^{3+}$ redox pairs in the olivine structure. The analysis of all cycles confirms the reversibility of this process. Additionally, an interesting shape of a peak associated with manganese oxidation was observed in the first cycle. A similar shape was observed for Li${}_{x}$Fe${}_{1-y}$Mn${}_{y}$PO${}_{4}$ by J. Molenda et al. [8]. It is possible that there is some irreversible process in this area, because the shape of the peak changed in the second cycle, and it remained unchanged for the rest of the cycles. A redox peak at potential 3.9 V corresponds to Mn${}^{2+}$/Mn${}^{3+}$ redox pairs. The most intriguing is the occurrence of a strong irreversible reduction at 3.2 V and an increasing current above 4.5 V. While the first of these phenomena can be assigned to the influence of vanadium as in the works [27,28], the origin of the irreversible peak above 4.5 V is not clear. However, one should keep in mind that at this potential, the extraction of the last lithium ion from Li${}_{x}$V${}_{2}$(PO${}_{4}$)${}_{3}$ should occur [29].

### 3.5. Transmission Electron Microscopy

**Figure 8:**
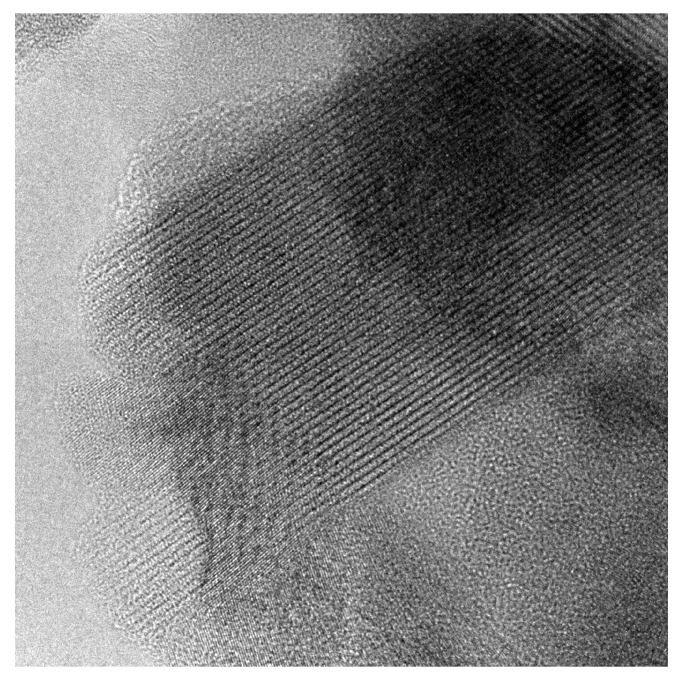
HR-TEM image of a sample with x=0.5 nanocrystallized at 480 °C. The visible area is 50 nm × 50 nm.

In Figure 8, a high-resolution TEM image of a nanograin is shown. One can see distinct crystallographic layers. The grain is surrounded with a residual glassy matrix. Such a microstructure is typical for materials synthesized in the way of the thermal nanocrystallization from a glass [30].

## 4. Conclusions

This research showed that thermal nanocrystallization of glassy analogs of LiFe${}_{1-x}$Mn${}_{x}$PO${}_{4}$ resulted in highly conducting materials that may be used as a cathode in Li-ion batteries. The addition of vanadium was proposed to improve its glass forming properties and to provide favorable conditions for electron hopping in the nanomaterials. The best electrical conductivity of the nanomaterial with x=0.5 exceeded 1 mS/cm. This giant and irreversible increase in the conductivity was explained with Mott’s electron hopping theory and a core-shell concept.

All of the three phases detected in the samples (i.e., LiFePO${}_{4}$, LiMnPO${}_{4}$, and Li${}_{3}$V${}_{2}$(PO${}_{4}$)${}_{3}$) are electrochemically active and therefore are suitable to be used as cathodes in Li-ion batteries, which was confirmed in preliminary galvanostatic and CV experiments. Further works on laboratory cells are worth carrying out in order to increase the electrochemical performance of the studied materials at higher current rates. Highly conducting material synthesized without carbon additives should be beneficial in terms of electrochemical performance under high current loads.

## Figures and Tables

**Table 1:** Nominal compositions of the samples under study.

*x*	Nominal Composition
0.25	Li(Fe${}_{0.75}$Mn${}_{0.25}$)${}_{0.88}$V${}_{0.08}$PO${}_{4}$
0.50	Li(Fe${}_{0.50}$Mn${}_{0.50}$)${}_{0.88}$V${}_{0.08}$PO${}_{4}$
0.75	Li(Fe${}_{0.25}$Mn${}_{0.75}$)${}_{0.88}$V${}_{0.08}$PO${}_{4}$

**Table 2:** Glass transition ($T_{g}$) and crystallisation ($T_{c}$) temperatures in the studied glassy samples, determined from DTA measurements carried out with 10 °C/min heating rate in argon flow. © The Electrochemical Society. Reproduced from Ref. [12] by permission of IOP Publishing. All rights reserved.

*x*	$T_{g}$ / °C	$T_{c1}$ / °C	$T_{c1^{\prime }}$ / °C	$T_{c2}$ / °C
0.25	434.3	498.2	—	555.7
0.50	433.9	492.0	—	561.9
0.75	435.4	490.4	536.0	586.6

## Data Availability

Not applicable.

## Abbreviations

The following abbreviations are used in this manuscript:

XRD	X-ray diffractometry
DTA	Differential thermal analysis
IS	Impedance spectroscopy
TEM	Transmission electron microscopy
